# Variations in home range and core area of red-backed voles (*Myodes regulus*) in response to various ecological factors

**DOI:** 10.1038/s41598-022-26779-7

**Published:** 2022-12-23

**Authors:** Jae-Kang Lee, Tae-Kyung Eom, Dong-Ho Lee, Hyeongyu Ko, Shin-Jae Rhim

**Affiliations:** School of Bioresource and Bioscience, Chung-Ang University, Ansung, 17546 South Korea

**Keywords:** Ecology, Zoology

## Abstract

The characteristics of animal distribution are determined by interactions between the resource requirements of animals and ecological factors. This study sought to evaluate the effects of diverse ecological factors on the home range and core area of red-backed voles (*Myodes regulus*) in a natural deciduous forest located on Mt. Gariwang, Pyeongchang and Jeongseon, South Korea. Our study focused on four types of ecological factors: topography, climate, cover, and demography. A total of 29 voles were radio-tracked from August to September 2021. Home range (95% utilization distribution; UD) and core area (50% UD) were calculated using the kernel density estimator (KDE). The home range (1659.49 m^2^) and core area (317.08 m^2^) were negatively affected by altitude. The lunar phase and temperature negatively and positively influenced the home range and core area, respectively. The home range was positively affected by understory vegetation, whereas the core area was not. The core area increased within microhabitats with a high density of conspecific individuals, with males having a larger home range (2006.19 m^2^) and core area (375.40 m^2^) than females (1043.13 m^2^ and 213.39 m^2^, respectively). These findings provide a deeper understanding of the diverse ecological factors affecting the distributions of animals, especially small rodents.

## Introduction

Knowledge of animal distribution is crucial for the management of invasive and endemic species and disease transmission within various ecosystems^1,2^. The distribution of wildlife is largely determined by the responses available in a given area, with individuals favoring locations where they can find food, shelter, nesting grounds, and mates^3^. Therefore, animal distribution is not only shaped by the interactions between the resource requirements of each individual but also by a wide variety of ecological factors^4^. Furthermore, animal migration tends to balance the benefits of foraging and mating and the costs of predation and environmental risks^5^.

Home range is the area where an individual usually travels to acquire food, shelter, nesting ground, and mates, except for the occasionally visited area^6^. Within the home range, the individual frequently uses an area characterized by abundant food and shelter resources, and this area is known as core area^7^. Therefore, the home range and core area are related to the distribution of individual’s space use, namely utilization distribution (UD)^8,9^. In general, the home range and core area are quantitatively defined as 95% and 50% UD, respectively^10^. The location and extent of the home range and core area provide insights into the habitat requirements of a certain individual in time and space^11^. In turn, different ecological factors appears to affect the sizes of home range and core area because the core area contains more abundant resources improving foraging efficiency of animals compared with other sections within the home range^12^, although the core area size are strongly associated with the home range size^7^.

Ecological factors include topography, climate, cover, and demography. Topographic factors affect habitat resources through changes in ambient temperature, humidity, water availability, and soil conditions^13,14^. Climatic factors, especially temperature and precipitation, are key factors for plant growth, resulting in variations in habitat and food resources for animals^15,16^. Additionally, moonlight affects predation risk for nocturnal prey by regulating the hunting success of predators^17^. Cover factors directly influence the activity patterns of animals, population size, and body mass because they provide food and shelter resources for wildlife^18,19^. In terms of demographic factors, the activity patterns of animals are modulated by the intensity of intra- and interspecific competition for limited resources^20^.

Rodents (order Rodentia) are an extremely diverse taxon accounting for approximately 40% of all mammal species^21^. Moreover, the numbers of this order exhibit a wide range of body sizes and inhabit diverse ecosystems^22^. Therefore, rodents occupy the crucial position in the food chain (e.g., consumers, prey) and participate in several ecological functions (e.g., seed dispersal and diverse transmission) within diverse ecosystems^23,24^. Thus, understanding the ecology of rodents, including their population dynamics, interaction with surrounding environments, activity patterns, and space usage, is of great importance to maintaining ecosystem health.

Small rodents have a narrow home range, and therefore their distributions have been studied somewhat more extensively than those of other mammals^25^. Most of these studies have focused on the effects of demography, such as the population sizes of conspecifics and competitors, sex, and body mass, on the space use of rodents^4,20,26^. In addition, relationships between rodents and predators have been researched to explain variations in rodents' activity patterns related to predation risk^27^. Furthermore, several studies have also characterized the effects of seasonal factors on the distribution of rodents in relation to demographic factors^5,11,28^. However, topographic and cover factors, which are independent variables, have not been studied as much as other ecological factors^29–31^. Additionally, very few studies have comprehensively assessed the effects of various types of ecological factors on species distribution in South Korea.

This study sought to evaluate the effects of various types of ecological factors on the home range and core area of small rodents in a natural deciduous forest in South Korea. We targeted the red-backed vole (*Myodes regulus*) to perform radiotelemetry monitoring because this species is dominant in the study area^22^. Ecological factors were classified into four types: topography (altitude and slope gradient), climate (temperature, precipitation, night length, lunar phase, and cloud cover), cover (vegetation, downed trees, and stone coverage), and demography (number of *M. regulus* and competitors, sex, and body mass). The following were our main hypotheses: (1) the sizes of the home range and core area of *M. regulus* would become larger at higher altitudes; (2) the lunar phase and temperature affect on the space use of *M. regulus*; (3) the home range and core area of *M. regulus* are affected by shelter- and food-related variables, respectively; (4) the distribution of *M. regulus* is influenced by the number of conspecific individuals, sex, and body mass.

## Methods

### Study area

**Figure 1:**
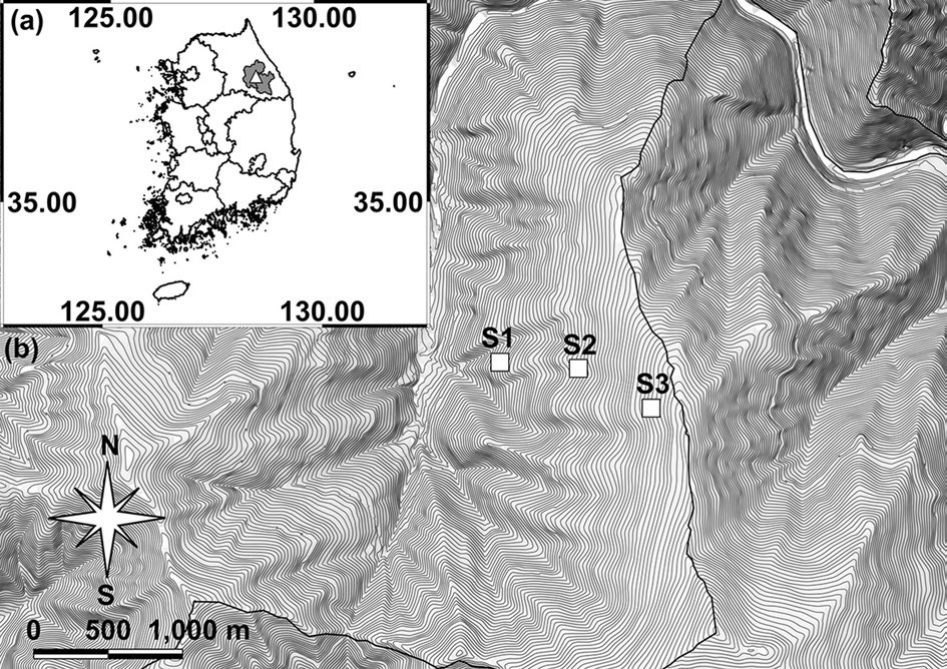
Location of the study area. (**a**) Location of Mt. Gariwang (white triangle) in Pyeongchang and Jeongseon (grey); (**b**) location of the three study sites (white box) in Mt. Gariwang. Both (**a**) and (**b**) were generated using QGIS 3.16 (http://www.qgis.org).

This study was conducted within a natural deciduous forest located on Mt. Gariwang (37°27.5′–37°30.5′ N, 128°30.5′–128°33.5′ E), Pyeongchang and Jeongseon, South Korea from May 2019 to September 2021 (Fig. 1a). The total area of Mt. Gariwang is approximately 40 km^2^, and the elevation is 1561 m above sea level (asl). The annual mean temperature and total precipitation in the study area were 11.2 °C (range: − 14.3–28.2 °C) and 1072.1 mm, respectively. The forests within this mountain consisted of temperate coniferous, deciduous, and mixed forests^32^. The study sites were located in a natural deciduous forest to minimize the effects of human activity on our results. Three study sites (100 × 100 m; 1 ha) were established at 900 m asl, 1,100 m asl, and 1,300 m asl on the same slope (Fig. 1b). A 7 × 7 grid with 15 m intervals (49 trapping points in total) was established within each of three sites. The study was carried out in compliance with the ARRIVE guidelines.

### Small rodent capture

Small rodents were captured using Sherman live traps (7.62 × 8.89 × 22.86 cm) placed at each of the 147 trapping points in the three study sites. In 2019 and 2020, small rodents were captured using the capture-mark-recapture (CMR) method from May to October for three consecutive nights each month. These traps were baited with peanuts. Each of the captured individuals was marked with a specific identification (ID) number using both toe clipping and ear punching. For each captured individuals, we recorded its species, sex, body mass, age structure, trapping point, and individual ID number.

In August 2021, adult *M. regulus* specimens were captured to attach transmitters. Pregnant or lactating females with obvious pregnancy, copulatory plug, and enlarged nipple were excluded from the radiotelemetry because the home range sizes of females were variable depending on different reproductive states^33^. We recorded the sex, body mass, age structure, and trapping location of each captured *M. regulus*. The captured adult *M. regulus* were anesthetized using an anesthesia box with 95% ethanol. Afterward, a very high frequency (VHF) tag (Ag317, 150 MHz, 0.4 g, 33 days, Lotek, Ontario, Canada) was fitted onto the neck of the captured individuals using a cable tie (0.8 g). The weight of the tag with the cable tie (1.2 g in total) was less than 5% of the body mass of the lightest individual (24.3 g) to ensure that the collar did not affect their activity^34^. The individuals were then released at the locations where they were captured after they had rested sufficiently in a recovery box supplied with food and water until we observed their normal behaviors without lethargy, shivering, or falls. The experimental protocols for the treatment and care of animals were reviewed and approved by the local ethics committee (Institutional Animal Care and Use Committee, Chung-Ang University; approval number: CAU 2019-00028). All methods were performed in accordance with the relevant guidelines and regulations.

### Home range and core area as dependent variables

We radio-tracked the radio-collared individuals from August 18 to September 17 using a Yagi antenna (Lotek, Ontario, Canada) and a receiver (Lotek, Ontario, Canada). The radio signals could be received up to 50 m under normal conditions. However, the distance which could receive the signals was variable depending on topography and the transmitter conditions. *M. regulus* is nocturnal, and therefore the radio-tracking was mostly carried out at night time divided into 8-h sessions (16:00–00:00, 20:00–04:00, and 00:00–08:00). The radio-collared individuals were located using the home-in technique^10^ and were tracked two times an hour per person. The tracked location was fixed using a handheld GPS device (Garmin GPSMAP; Garmin Ltd., Kansas, USA).

The home range and core area were estimated using the kernel density estimator (KDE) with the ‘adehabitatHR’ package in R^35,36^. First, the UD was calculated from all fixed coordinates for each individual. Afterward, we estimated the home range and core area using the UD depending on its occupancy frequency (95% and 50%, respectively). The estimated home range and core area data were transferred to QGIS 3.16 (QGIS Development Team) as a shapefile to extract each ecological factor value (except for climatic factors) corresponding to each home range and core area using zonal statistics.

### Topographic factors as independent variables

The topographic factors included altitude (meters above sea level) and slope gradient (°), both of which were independent variables. Digital maps (1:25,000) covering Mt. Gariwang were downloaded from the national geographic information institute (NGII) website (https://map.ngii.go.kr). A digital elevation model (DEM) with a 5 × 5 m pixel resolution was generated using the digital maps in QGIS. Afterward, we calculated the mean altitude and slope gradient of each home range and core area using zonal statistics.

### Climatic factors as independent variables

The climatic factors evaluated in this study included temperature (℃), precipitation (mm), night length (hours), lunar phase (%), and cloud cover (%). Temperature data were recorded using three HOBO data loggers at 1-h intervals (Onset Computer Corporation, Massachusetts, USA) at the center of each study site. Precipitation, night length, and cloud cover in the study area were downloaded from the Korean meteorological administration (KMA) national climate data center website (https://data.kma.go.kr). Lunar phase data were obtained from the Korean astronomy and space science institute (KASSI) website (https://astro.kasi.re.kr). The mean values of the climatic data corresponding to time (temperature, precipitation, and cloud cover) or date (night length and lunar phase) were calculated during the recording of each radio-tracked location.

### Cover factors as independent variables

Microhabitat conditions such as cover factors were surveyed from July and August in 2019 and 2020. Here, we measured ground vegetation coverage (0–1 m height), understory vegetation coverage (1–2 m height), stone coverage, number of standing trees (n/ha), basal area (m^2^/ha), and number and volume of downed trees (n/ha and m^3^/ha, respectively) within a circle with a 5.64 m radius centered on each of the trapping points. The coverage of ground vegetation, understory vegetation, and stones were categorized into four levels as follows: 0 (coverage = 0%), 1 (1–33%), 2 (34–66%), and 3 (> 67%)^37^. The measurements were imported into QGIS and rasterized into grids covering the study sites with a 5 × 5 m pixel resolution. Afterward, we calculated the mean values of each factor corresponding to the home range and core area using zonal statistics.

### Demographic factors as independent variables

The Demographic factors examined in this study included the number of individuals, sex, and body mass. We captured three species, 175 individuals of red-backed vole (*Myodes regulus*), 96 individuals of striped field mouse (*Apodemus agrarius*), and 136 individuals of Korean field mouse (*A. peninsulae*) within the study sites from May to October 2019 and 2020. The aforementioned data was imported into QGIS and rasterized into the grids used in the rasterization of cover factors. Afterward, we calculated the mean values of the number of individuals of each species corresponding to each home range and core area using zonal statistics. The data for sex and body mass was recorded when the radio-collared individuals were captured.

### Statistical analysis

The normality of all independent variables was assessed using the Shapiro–Wilk test, after which the multicollinearity between variables belonging to each of the four types of ecological factors was removed via Spearman correlation analysis. When a highly correlated pair (Spearman’s coefficient > 0.7) of two variables was identified, one variable of the pair was discarded based on its ecological relevance or correlation with home range and core area^38^. The slope gradient of the core area and night length were eliminated in this procedure.

Four linear models (LMs) were constructed to evaluate the effect of each of the four types of ecological factors on the space use of *M. regulus* using the ‘stats’ package in R^35^. The global models of the four LMs were as follows: home range ~ altitude + slope gradient, core area ~ altitude (topographic factors); home range or core area ~ temperature + precipitation + lunar phase*cloud cover (climatic factors); home range or core area ~ ground vegetation + understory vegetation + stone + number of standing trees + basal area + number of downed trees + volume of downed trees (cover factors); home range or core area ~ number of *M. regulus* + number of *A. agrarius* + number of *A. peninsulae* + sex + body mass (demographic factors). The best models among the candidate models for each procedure were selected based on the Akaike information criterion with corrections for small samples (AIC*c*).

## Results

A total of 29 adult *M. regulus* specimens were radio-tagged, out of which 4 individuals could not be radio-tracked due to one migration, one predation, and two unknown cases. Out of the remaining 25 individuals (16 male and 9 female), 7 individuals were captured at 900 m asl, 10 at 1100 m asl, and 8 at 1300 m asl. The body mass of these individuals ranged from 24.3 g to 34.1 g (28.66 ± 0.49; mean ± SE). The mean number of fixes was 37.68 ± 1.26 per individual. The mean duration of radio-tracking for each individual was 6.72 ± 0.31 days. The mean sizes of the home range and core area were 1659.49 ± 252.06 m^2^ and 317.08 ± 50.21 m^2^, respectively. The mean home range and core area of males were 2006.19 ± 344.57 m^2^ and 375.40 ± 67.20 m^2^, whereas females were 1043.13 ± 243.94 m^2^ and 213.39 ± 62.11 m^2^, respectively.

### Topographic effects on home range and core area

**Figure 2:**
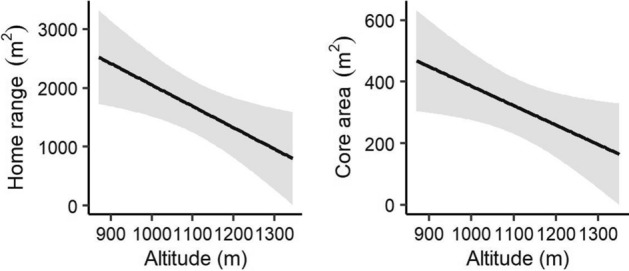
Topographic effects on the home range and core area of red-backed voles (*Myodes regulus*) determined by the linear models.

Altitude was among the best models of home range and core area (Table 1). Therefore, the slope gradient did not have any effect on the distribution of *M. regulus*. The home range and core area of *M. regulus* narrowed with increasing altitude (home range: β = − 3.63, SE = 1.41, t = − 2.58, *p* = 0.02; core area: β = − 0.63, SE = 0.29, t = − 2.18, *p* = 0.04; Fig. 2).

**Table 1:** Linear models that most accurately explained the effects of different ecological factors on the distribution of red-backed voles (*Myodes regulus*).

Distribution	Ecological factors	Models	*k*	AICc	w_i_
Home range	Topography	[Intercept + alt]	3	427.69	0.61
	Climate	[Intercept + lp]	3	428.35	0.18
	Cover	[Intercept + uv]	3	429.59	0.11
	Demography	[Intercept + n_mr + sex]	4	429.54	0.15
Core area	Topography	[Intercept + alt]	3	2688.05	0.46
	Climate	[Intercept + tem]	3	348.34	0.20
	Cover	[Intercept]	2	350.76	0.10
	Demography	[Intercept + n_mr + sex]	4	348.71	0.16

alt, altitude; lp, lunar phase; tem, temperature; uv, understory vegetation; n_mr, number of *Myodes regulus.*

### Climatic effects on home range and core area

**Figure 3:**
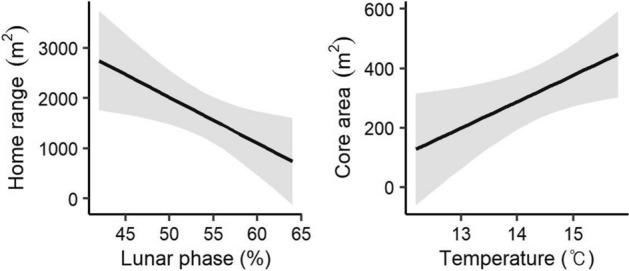
Climatic effects on the home range and core area of red-backed voles (*Myodes regulus*) determined by the linear models.

The lunar phase was also among the best models of home range, whereas temperature was among the best models for core area (Table 1). Lunar phase had a negative effect on the home range of *M. regulus* (β = − 91.16, SE = 37.64, t = − 2.42, *p* = 0.02; Fig. 3). Moreover, the size of the core area increased with higher temperatures (β = 88.81, SE = 39.27, t = 2.26, *p* = 0.03).

### Cover effects on home range and core area

**Figure 4:**
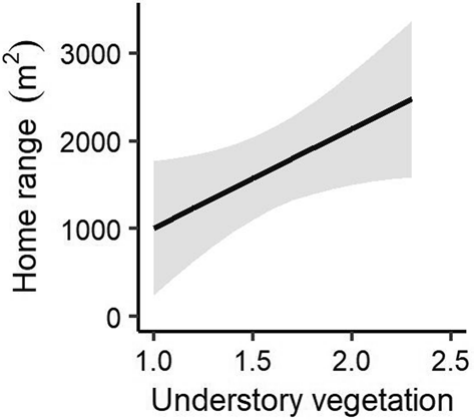
Cover effects on the home range of red-backed voles (*Myodes regulus*) determined by the linear model.

The best home range model included the understory vegetation (Table 1). However, the best model of core area was a null model, and therefore the cover factors did not have any effects on the core area. The home range of *M. regulus* widened as understory vegetation became more abundant (β = 1133.50, SE = 536.50, t = 2.11, *p* = 0.05; Fig. 4).

### Demographic effects on home range and core area

**Figure 5:**
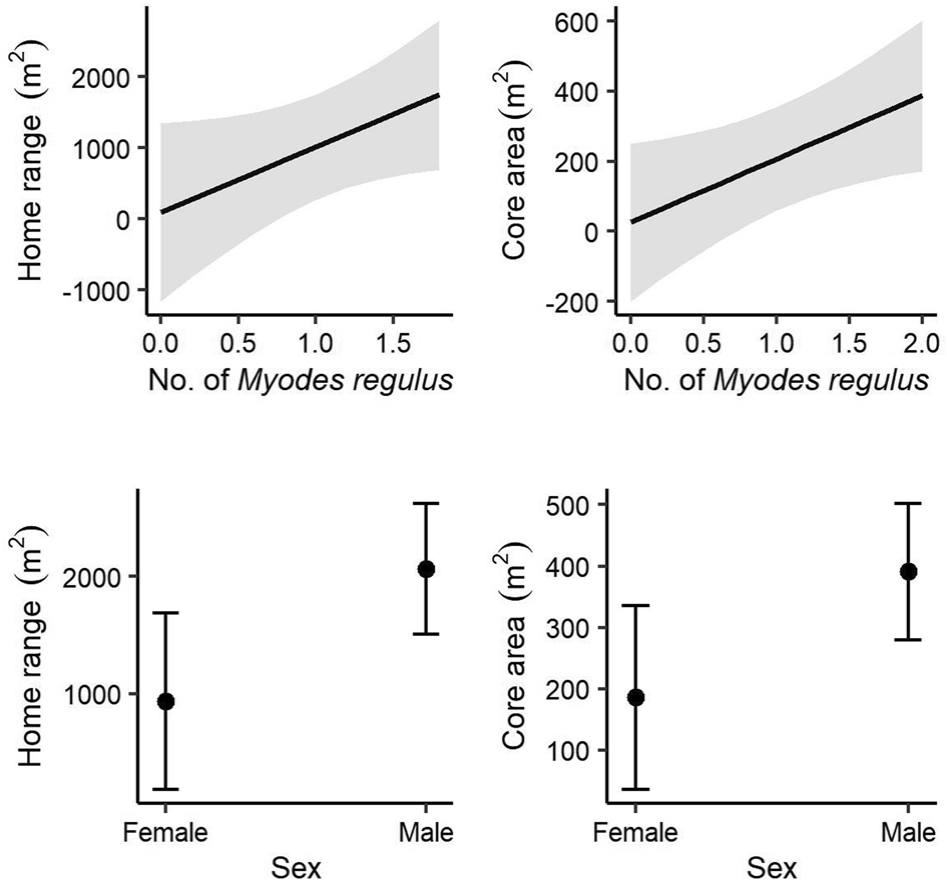
Demographic effects on the home range and core area of red-backed voles (*Myodes regulus*) determined by the linear models.

The best models of home range and core area included the number of *M. regulus* and sex (Table 1). Moreover, home range was not influenced by the number of *M. regulus* (β = 915.23, SE = 498.06, t = 1.84, *p* = 0.08; Fig. 5), and core area broadened within microhabitats highly inhabited by *M. regulus* (β = 180.83, SE = 83.17, t = 2.17, *p* = 0.04). Male of *M. regulus* had wider home range sizes (β = 1133.04, SE = 482.50, t = 2.35, *p* = 0.03) and core area (β = 205.51, SE = 96.15, t = 2.14, *p* = 0.04) than females.

## Discussion

Our study demonstrated the effects of four types of ecological factors on the distribution of *M. regulus*. Regarding the topographic factors, the sizes of the home range and core area decreased at higher altitudes. In terms of the climatic factors, a brighter lunar phase resulted in a narrow home range, whereas a high temperature induced a broad core area. The cover factors influenced only the home range, and the size of the home range was increased within microhabitats with abundant understory vegetation. Lastly, regarding the demographic factors, male *M. regulus* exhibited a wider distribution than females, and the core area widened within microhabitats abundantly inhabited by *M. regulus*.

We hypothesized that the sizes of the home range and core area would increase with altitude because habitats at high altitudes have fewer food resources than at low altitudes^22^, which causes the animals to travel farther to find adequate food^39^. Unexpectedly, the sizes of the home range and core area of *M. regulus* were smaller at high altitudes than at low altitudes in our study. The radio-tracking survey was performed from mid-August to mid-September when there was a season transition from summer to fall. This period is characterized by an abrupt decrease in temperature, and low-temperature stress was more accentuated at high altitudes. Small rodents become less physically active at low temperatures because they minimize their exposure to extreme conditions^5^. Therefore, the lower temperatures at high altitudes appeared to significantly decrease the home range and core area of *M. regulus*.

The lack of shelter resources was likely another reason for the decreasing distribution of *M. regulus* at higher altitudes. Understory vegetation (e.g., shrub) plays a key role as a shelter resource for small rodents^40^. However, Lee et al.^22^ reported out that the understory vegetation decreased with higher altitudes in the study area. Therefore, our findings suggested that high-altitude sites lacked adequate shelters, which likely increased the predation risk for this species, thus reducing the activity of *M. regulus*.

The size of the home range was influenced by the lunar phase, whereas the size of the core area was affected by temperature in this study. Nocturnal animals regulate their activities depending on variety of climatic factors such as temperature, precipitation, and moonlight^41^. In this respect, a brighter lunar phase (e.g., full moon) would enable predators to easily spot their prey at night, thus increasing the predation risks for many nocturnal prey species including small rodents^42^. Small rodents are less active and more vigilant under this condition^43,44^. In our study area, the main nocturnal predators are owls, like eagle owl (*Bubo Bubo*) and tawny owl (*Strix aluco*). These owls are more active with increasing moonlight because nocturnal vision is enhanced under bright moonlight conditions^45,46^. The home range is the whole area where an individual lives and includes some zones with deficient available resources. The activity of *M. regulus* appeared to be restricted in these zones under bright moonlight due to increased predation risk. In contrast with the home range, the core area is a specific location where available resources are abundant. Given that *M. regulus* individuals must actively explore their home range in search for food, a high temperature appeared to help *M. regulus* easily forage without physically stressful conditions^5^, resulting in a wider core area.

We hypothesized that the home range and core area of *M. regulus* are respectively affected by shelter- and food-related variables because the core area within the home range is highly related to the quality and quantity of food resources^47^. In this study, the understory vegetation (a cover factor) affected the size of the home range of *M. regulus*, whereas no cover factors influenced the size of the core area. Shrubs are important components of the understory vegetation that provide shelter resources and reduce predation risk for small rodents^38,48^. Therefore, microhabitats with dense understory vegetation appeared to offer sufficient shelter and corridor resources for *M. regulus*, thus enabling this species to widely disperse under lower predation risk. We did not identify any cover factors affecting the core area of *M. regulus*. Voles are known to consume the bark and roots of young trees when food is scarce^49^. Moreover, *M. regulus* prefers various types of plant seeds, which we did not consider in this study^50^. Therefore, future studies should account for other food-related variables to evaluate the effects of vegetation cover on the home range and core area of small rodents.

The core area of *M. regulus* was affected by the number of conspecific individuals, whereas the home range was not. This study was conducted at the end of the breeding season of small rodents in South Korea^50^. In this transition period from breeding season to non-breeding season, *Myodes* species change their spatial behavior from which males seek mates and females care their young to which both males and females forage for their own maintenances^51,52^. Therefore, the *M. regulus* population did not have to compete with conspecifics for mating within their home range, and thus the number of conspecific individuals did not appear to affect their home range. In contrast with the home range, given that small rodents must consistently gather food in preparation for the winter^50,53^, *M. regulus* appeared to occupy a broad core area to improve its odds of findings adequate food sources within regions with a high density of conspecifics. The home range and core of male voles were wider than those of females, which was consistent with the findings of previous studies^1,54^. In breeding season, *Myodes* species show differences in spatial distributions between male and female because of different reproductive strategies between sex^4^. The space use of male overlaps with several males and females, whereas females show territorial behavior to achieve sexual maturity and successful reproduction^55,56^. However, our study period corresponded with the transition period from breading season to non-breading season. In this transition period, females’ change in spatial behavior would be delayed more than that of males’ in order to rear their young until the young disperse from them^57^. Therefore, this result appeared to be related to the maternal nesting behavior, whereby females remain closer to their nest to defend their young, as with the southern red-backed vole *(M. gapperi*) which females had a narrower home range (0.37 ha) and core area (0.12 ha) than males (2.11 ha and 0.70 ha, respectively)^4^. We expected that the sizes of the home range and core area of *M. regulus* would vary depending on the individual’s body mass. However, this was not the case. This appeared to be due to the overall decrease in the home range and core area of *M. regulus* during the radio-tracking period. The period of the radio-tracking survey in this study coincided with the time when the ambient temperature decreased dramatically. This stressful condition restricts the overall activities of small rodents^58,59^. Therefore, it appears that temperature stress offsets the influence of body mass on the distribution of *M. regulus*. However, additional studies covering other seasons are needed to confirm this phenomenon.

Our findings demonstrated that the sizes of the home range and core area of *M. regulus* were affected by various ecological factors and the influence of these factors on the home range and core area also exhibited distinct variations. Among the evaluated topographic factors, altitude strongly affected the home range and core area, which was most likely due to altitude-related variations in temperature and shelter resources. Regarding the climatic factors, the home range and core area were regulated by the lunar phase and the temperature, respectively. These two variables appeared to be related to predation risk and foraging efficiency, respectively. Among the vegetation cover factors, the understory vegetation only influenced the home range due to its role as a shelter resource for *M. regulus*. Neither of the evaluated cover factors were found to affect the core area. However, it appears that other variables such as the occurrence of young trees and seed abundance could affect the core area of *M. regulus*. In terms of demographic factors, the number of conspecific individuals appeared to extend the core area of *M. regulus*, as covering a larger area would presumably provide a competitive advantage and increase the likelihood of finding food. Furthermore, *M. regulus* females exhibited a narrower home range and core area compared to males, which was most likely due to maternal/nesting behaviors. These findings contribute to a deeper understanding of the diverse ecological factors affecting the variations in the home range and core area of rodents, particularly *M. regulus*. However, the duration of this study was relatively short and therefore did not consider seasonal variables that likely have a significant impact on small rodent distribution, as discussed above. Therefore, a long-term monitoring study covering other seasons and diverse potential variables is needed to increase our knowledge of the ecological factors that determine the spatiotemporal distribution of small rodent.

## Data Availability

The datasets used and/or analysed during the current study available from the corresponding author on reasonable request.

## References

[1] Gorosito I, Benitez A, Busch M (2020). Home range variability, spatial aggregation, and excursions of *Akodon*
*azarae* and *Oligoryzomys*
*flavescens* in Pampean agroecosystems. Integr. Zool..

[2] Christy MT, Savidge JA, Adams AAY, Gragg JE, Rodda GH (2017). Experimental landscape reduction of wild rodents increases movements in the invasive brown treesnake (*Boiga **irregularis*). Manag. Biol. Invasion..

[3] Cutrera AP, Antinuchi CD, Mora MS, Vassallo AI (2006). Home-range and activity patterns of the south American subterranean rodent *Ctenomys*
*talarum*. J. Mammal..

[4] Tisell HB, Degrassi AL, Stephens RB, Rowe RJ (2019). Influence of field technique, density, and sex on home range and overlap of the southern red-backed vole (*Myodes **gapperi*). Can. J. Zool..

[5] Vieira EM, Baumgarten LC, Paise G, Becker RG (2010). Seasonal patterns and influence of temperature on the daily activity of the diurnal neotropical rodent *Necromys*
*lasiurus*. Can. J. Zool..

[6] Burt WH (1943). Territoriality and home range concepts as applied to mammals. J. Mammal..

[7] Samuel MD, Pierce D, Garton EO (1985). Identifying areas of concentrated use within the home range. J. Anim. Ecol..

[8] Worton B (1989). Kernel methods for estimating the utilization distribution in home-range studies. Ecology.

[9] Powell RA, Mitchell MS (2012). What is a home range?. J. Mammal..

[10] White GC, Garrott RA (1990). Analysis of Wildlife Radio-tracking Data.

[11] Lee EJ, Rhim SJ, Lee WS (2012). Seasonal movements and home range sizes of Korean field mouse *Apodemus*
*peninsulae* in unburned and post-fire pine planted stands within a pine forest. J. Anim. Vet. Adv..

[12] Thompson RL, Chambers CL, McComb BC (2009). Home range and habitat of western red-backed voles in the Oregon Cascades. Northwest Sci..

[13] Tu CL, He TB, Lu XH, Luo Y, Smith P (2018). Extent to which pH and topographic factors control soil organic carbon level in dry farming cropland soils of the mountainous region of Southwest China. CATENA.

[14] Khandelwal S, Goyal R, Kaul N, Mathew A (2018). Assessment of land surface temperature variation due to change in elevation of area surrounding Jaipur, India. Egypt. J. Remote Sens..

[15] Hatfield JL, Prueger JH (2015). Temperature extremes: Effect on plant growth and development. Weather Clim. Extrem..

[16] Fang JY (2005). Precipitation patterns alter growth of temperate vegetation. Geophys. Res. Lett..

[17] Palmer MS, Fieberg J, Swanson A, Kosmala M, Packer C (2017). A 'dynamic' landscape of fear: Prey responses to spatiotemporal variations in predation risk across the lunar cycle. Ecol. Lett..

[18] Lee JK, Hwang HS, Eom TK, Rhim SJ (2018). Influence of tree thinning on abundance and survival probability of small rodents in a natural deciduous forest. Turk. J. Zool..

[19] Navarro-Castilla A, Barja I (2019). Stressful living in lower-quality habitats? Body mass, feeding behavior and physiological stress levels in wild wood mouse populations. Integr. Zool..

[20] Casula P, Luiselli L, Amori G (2019). Which population density affects home ranges of co-occurring rodents?. Basic Appl. Ecol..

[21] D'Elia G, Fabre PH, Lessa EP (2019). Rodent systematics in an age of discovery: Recent advances and prospects. J. Mammal..

[22] Lee JK, Eom TK, Bae HK, Lee DH, Rhim SJ (2022). Responsive strategies of three sympatric small rodents to the altitudinal effects on microhabitats. Anim. Biol..

[23] Lee JK, Hwang HS, Eum TK, Bae HK, Rhim SJ (2020). Cascade effects of slope gradient on ground vegetation and small-rodent populations in a forest ecosystem. Anim. Biol..

[24] Orrock JL, Connolly BM (2016). Changes in trap temperature as a method to determine timing of capture of small mammals. PLoS ONE.

[25] Endries MJ, Adler GH (2005). Spacing patterns of a tropical forest rodent, the spiny rat (*Proechimys*
*semispinosus*), in Panama. J. Zool..

[26] Kawata M, Saitoh T (1988). The effect of introduced males on spatial patterns of initially introduced red-backed voles. Acta Theriol..

[27] Desy E, Batzli G, Liu J (1990). Effects of food and predation on behaviour of prairie voles: A field experiment. Oikos.

[28] Attuquayefio D, Gorman M, Wolton R (1986). Home range sizes in the wood mouse *Apodemus*
*sylvaticus*: Habitat, sex and seasonal differences. J. Zool..

[29] Lovari S, Sforzi A, Mori E (2013). Habitat richness affects home range size in a monogamous large rodent. Behav. Process..

[30] Puckey H, Lewis M, Hooper D, Michell C (2004). Home range, movement and habitat utilisation of the Carpentarian rock-rat (*Zyzomys*
*palatalis*) in an isolated habitat patch. Wildlife Res..

[31] Jones EN (1990). Effects of forage availability on home range and population density of *Microtus **pennsylvanicus*. J. Mammal..

[32] Chun JH, Ali A, Lee CB (2020). Topography and forest diversity facets regulate overstory and understory aboveground biomass in a temperate forest of South Korea. Sci. Total. Environ..

[33] Koskela E, Mappes T, Ylonen H (1997). Territorial behaviour and reproductive success of bank vole *Clethrionomys*
*glareolus* females. J. Anim. Ecol..

[34] Vlasata T (2017). Daily activity patterns in the giant root rat (*Tachyoryctes** macrocephalus*), a fossorial rodent from the Afro-alpine zone of the Bale Mountains, Ethiopia. J. Zool..

[35] R Core Team (2013). R: A Language and Environment for Statistical Computing.

[36] Calenge C (2006). The package "adehabitat" for the R software: A tool for the analysis of space and habitat use by animals. Ecol. Model..

[37] Rhim SJ, Kim KJ, Son SH, Hwang HS (2012). Effect of forest road on stand structure and small mammals in temperate forests. J. Anim. Vet. Adv..

[38] Carrilho M, Teixeira D, Santos-Reis M, Rosalino LM (2017). Small mammal abundance in Mediterranean Eucalyptus plantations: How shrub cover can really make a difference. For. Ecol. Manag..

[39] Emsens WJ (2013). Effects of food availability on space and refuge use by a beotropical scatterhoarding rodent. Biotropica.

[40] Malo AF (2013). Positive effects of an invasive shrub on aggregation and abundance of a native small rodent. Behav. Ecol..

[41] Johnson MD, De Leon YL (2015). Effect of an invasive plant and moonlight on rodent foraging behavior in a coastal dune ecosystem. PLoS ONE.

[42] Mori E, Sangiovanni G, Corlatti L (2020). Gimme shelter: The effect of rocks and moonlight on occupancy and activity pattern of an endangered rodent, the garden dormouse *Eliomys*
*quercinus*. Behav. Process..

[43] Prugh LR, Golden CD (2014). Does moonlight increase predation risk? Meta-analysis reveals divergent responses of nocturnal mammals to lunar cycles. J. Anim. Ecol..

[44] Orrock JL, Danielson BJ, Brinkerhoff RJ (2004). Rodent foraging is affected by indirect, but not by direct, cues of predation risk. Behav. Ecol..

[45] Penteriani V, Delgado MDM, Campioni L, Lourenco R (2010). Moonlight makes owls more chatty. PLoS ONE.

[46] Penteriani V, Kuparinen A, del Mar Delgado M, Lourenço R, Campioni L (2011). Individual status, foraging effort and need for conspicuousness shape behavioural responses of a predator to moon phases. Anim. Behav..

[47] Reher S, Dausmann KH, Warnecke L, Turner JM (2016). Food availability affects habitat use of Eurasian red squirrels (*Sciurus vulgaris*) in a semi-urban environment. J. Mammal..

[48] Lee JK, Hwang HS, Eom TK, Lee DH, Rhim SJ (2022). Slope gradient effect on microhabitat and small rodents in a tree thinned Japanese larch plantation. Pak. J. Zool..

[49] Heroldova M, Bryja J, Janova E, Suchomel J, Homolka M (2012). Rodent damage to natural and replanted mountain forest regeneration. Sci. World J..

[50] Jo YS, Baccus JT, Koprowski JL (2018). Mammals of Korea.

[51] Bondrup-Nielsen S (1986). Investigation of spacing behavior of *Clethrionomys*
*gapperi* by experimentation. J. Anim. Ecol..

[52] Ylonen H, Kojola T, Viitala J (1988). Changing female spacing behavior and demography in an enclosed breeding population of *Clethrionomys*
*glareolus*. Holarctic Ecol..

[53] Vander Wall SB (2019). Seed harvest by scatter-hoarding yellow pine chipmunks (*Tamias **amoenus*). J. Mammal..

[54] Lee EJ, Rhim SJ (2016). Seasonal home ranges and activity of three rodent species in a post-fire planted stand. Folia Zool..

[55] Bondrup-Nielsen S, Ims RA (1986). Reproduction and spacing behavior of females in a peak density population of *Clethrionomys*
*glareolus*. Holarctic Ecol..

[56] Bujalska G, Grum L (1989). Social organization of the bank vole (*Clethrionomys*
*glareolus*, Schreber 1780) and its demographic consequences: A model. Oecologia.

[57] Henttonen H (2022). Importance of demography in understanding disease ecology in small mammals. Therya.

[58] Rezende EL, Cortes A, Bacigalupe LD, Nespolo RF, Bozinovic F (2003). Ambient temperature limits above-ground activity of the subterranean rodent *Spalacopus cyanus*. J. Arid Environ..

[59] Guiden PW, Orrock JL (2020). Seasonal shifts in activity timing reduce heat loss of small mammals during winter. Anim. Behav..

